# Inhibition of Ephrin-B2 in brain pericytes decreases cerebral pathological neovascularization in diabetic rats

**DOI:** 10.1371/journal.pone.0210523

**Published:** 2019-01-08

**Authors:** Maha Coucha, Amy C. Barrett, Mostafa Elgebaly, Adviye Ergul, Mohammed Abdelsaid

**Affiliations:** 1 Department of Pharmaceutical Sciences, School of Pharmacy, South University, Savannah, Georgia, United States of America; 2 Biomedical Sciences Department, School of Medicine, Mercer University, Savannah, Georgia, United States of America; 3 Department of Pharmaceutical Sciences, College of Pharmacy, Larkin University, Miami, Florida, United States of America; 4 Charlie Norwood VA Medical Center, Augusta, Georgia, United States of America; 5 Department of Physiology, Medical College of Georgia, Augusta University, Augusta, Georgia, United States of America; Cedars-Sinai Medical Center, UNITED STATES

## Abstract

We have previously shown that diabetes causes dysfunctional cerebral neovascularization that increases the risk for cerebrovascular disorders such as stroke and cognitive impairment. Pericytes (PCs) play a pivotal role in the angiogenic process through their interaction with the endothelial cells (EC). Yet, the role of PCs in dysfunctional cerebral neovascularization in diabetes is unclear. In the present study, we tested the hypothesis that the increased proangiogenic Ephrin-B2 signaling in PCs contributes to the dysfunctional cerebral neovascularization in diabetes. Type-II diabetes was induced by a combination of high fat diet and low dose streptozotocin injection in male Wistar rats. Selective in vivo Ephrin-B2 silencing in brain PCs was achieved using the stereotactic injection of adeno-associated virus (AAV) with NG2-promoter that expresses Ephrin-B2 shRNA. Neovascularization was assessed using vascular fluorescent dye stain. Novel object recognition (NOR) test was used to determine cognitive functions. Human brain microvascular pericytes HBMVPCs were grown in high glucose 25 mM and palmitate 200 uM (HG/Pal) to mimic diabetic conditions. Scratch migration and tube formation assays were conducted to evaluate PC/EC interaction and angiogenic functions in PC/EC co-culture. Diabetes increased the expression of Ephrin-B2 in the cerebrovasculature and pericytes. Concomitant increases in cerebral neovascularization parameters including vascular density, tortuosity and branching density in diabetic rats were accompanied by deterioration of cognitive function. Inhibition of Ephrin-B2 expression in PCs significantly restored cerebral vascularization and improved cognitive functions. HG/Pal increased PC/EC angiogenic properties in co-culture. Silencing Ephrin-B2 in PCs significantly reduced PC migration and PC/EC co-culture angiogenic properties. This study emphasizes the significant contribution of PCs to the pathological neovascularization in diabetes. Our findings introduce Ephrin-B2 signaling as a promising therapeutic target to improve cerebrovascular integrity in diabetes.

## Introduction

Diabetes causes devastating vascular complications such as diabetic retinopathy, stroke and cognitive impairment [1]. The cost for managing diabetes-induced vascular complications is rising every day in the United States and worldwide with the increasing numbers of diabetic patients [2, 3]. Therefore, there is an urgent need to understand how diabetes affects the cerebral microvasculature, a common player in most diabetes-induced cerebral complications.

Emerging evidence suggests that diabetes mediates pathological cerebrovascular neovascularization, which is correlated with cognitive dysfunction [4–6]. Limited understanding of the underlying mechanisms by which diabetes alters cerebrovascular architecture is a fundamental barrier to the development of preventive and therapeutic strategies. The purpose of the current study is to unravel the role of Ephrin-B2 signaling in pericytes as one of the signaling mechanisms that is involved in diabetes-mediated pathological cerebrovascular neovascularization.

Pericytes are uniquely positioned cells that are embedded in the basement membrane tightly wrapped around the endothelial cells (ECs) [7, 8]. Moreover, pericytes have long processes that encircle the micro vessels and they are important for maintaining vascular stability and integrity [9, 10]. It has long been thought that pericytes provide only as a scaffolding function, but it is now accepted that pericytes have a pivotal role in endothelial cell guidance and angiogenesis [11–14]. Yet, the role of pericytes in diabetes-induced cerebral microangiopathy remains a big gap in our knowledge.

The Ephrin/Eph system is the largest family of tyrosine kinase receptors involved in physiologic and pathologic angiogenesis [15–17]. Ephrin-B2 and its receptor EphB4 play a crucial role in the development and postnatal angiogenesis [15, 16]. Ephrin-B2 is expressed in both endothelial and pericyte cells. Studies showed that Ephrin-B2 is important for cell-cell communication [16, 18]. Yet, how diabetes alters Ephrin-B2 expression in pericytes in the cerebrovasculature is unknown.

Taken all together, in this study we hypothesize that pericytes play a crucial role in diabetes-mediated pathological cerebral neovascularization via dysregulation of Ephrin-B2 signaling.

## Methods

### Animals

Experiments were performed using 4-weeks old male Wistar rats (Charles River, Wilmington, MA). The animals were housed at the Mercer University animal care facility that is approved by the American Association for Accreditation of Laboratory Animal Care. All protocols were approved by Mercer University Institution Animal Care And Use Committee. Type II diabetes was induced using a low dose of streptozotocin (35 mg/kg body weight, Alfa Aesar, Tewksbury, MA) followed by 8 weeks of high fat diet (45 kcal% fat, Research Diets Inc., New Brunswick, NJ). Control animals were fed standard rat chow (10 kcal% fat, Research Diets Inc., New Brunswick, NJ) and tap water ad libitum. Animals were monitored daily for any sign of distress. Body weights and blood glucose measurements were taken weekly. Animals were excluded if their body weights drops by 15% of initial body weight. Blood glucose (BG) measurements were taken from tail vein samples using a commercially available glucometer (Freestyle, Abbott Diabetes Care, Inc., Alameda, CA). At the end of the study, control rats body weights were 397.4±36.2 gm, while diabetic’s 335.7±53.12 gm. Average BG levels were 117.8±8.17 and 228±33.6 mg/dl for control and diabetic animals, respectively.

Diabetic animals were divided into 2 groups, one group was injected adenovirus with NG2 promoter that expresses green fluorescence protein (GFP) and Ephrin-B2 shRNA (ID: VB160513-1081jgm-pAAV[Exp]-NG2enhancerMinPro>EGFP:5'miR30:rEfnb2[shRNA#8]:3'miR30, Vector builder, Cyagen Biosciences Inc., Santa Clara, CA). 3 μl of purified viral particles (1× 10^11^ GC/ml) were injected over ten min via a 30-gauge needle into the striatum at stereotactic coordinates (bregma: AP– 0.5, ML– 1 mm, DV– 4 mm). The animals were anesthetized with Ketamine (100 mg/kg body weight) and Xylazine (10 mg/kg body weight) mixture. Delayed release buprenorphine (1.2 mg/kg body weight) was given SQ before stereotactic injection to minimize animal suffering and distress. For sacrifice, animals were deeply anesthetized using isoflurane until the hind toe pinch is ablated before decapitation. Selective inhibition of Ephrin-B2 expression in pericytes was confirmed by Western blot analysis and immunohistochemical localization of GFP expression and pericyte marker 2 weeks after injection.

### Assessment of neovascularization parameters

Vascularization patterns and density were measured using the direct labeling and visualization of blood vessels with lipophilic carbocyanine dye DiI (Dil: 1,1’-dioctadecyl-3,3,3’,3’-tetramethylindocarbocyanine perchlorate, D-282, Invitrogen/Molecular Probes, Carlsbad, CA). Briefly, animals were anesthetized and perfused with ice cold PBS buffer followed by 30 ml of diluted Dil dye working solution [19]. Brains were cut into 2-mm slices (labeled A–G rostral to caudal). Z-stacked confocal images of 50-100-μm sections from regions C (medial where the MCA branches out to supply the frontal motor cortex, bregma 1 to −1) were acquired using Nikon A confocal microscope. Regions of interest (ROIs), within the striatum, were based on our previous findings [20]. Vascular density, branching density and tortuosity index were calculated using FIJI software, an image processing and analysis version of the ImageJ software. Vascular density refers to the density of Dil-stained vasculature from the merged planes over the total number of planes in the section. Branching density was calculated as ratio between the numbers of branches over the longest shortest branch path. For vessel tortuosity, tortuosity index was calculated as the ratio between branch length and euclidian distance of the branch. ROIs measurement from one animal comprised a mean value of six images from striatal region.

### Immuno-localization studies

Brain sections were blocked using 0.1% horse serum dissolved in 1% BSA in 0.3% Triton X-100 in phosphate buffer. Sections were reacted to polyclonal anti- Ephrin-B2 antibody (R&D systems; Minneapolis, MN). Primary antibodies were specific for rat and used in 1:200 dilutions in 1% BSA in 0.3% Triton X-100 in phosphate buffer. Sections were then incubated with 1:1000 dilutions of goat anti-rabbit antibodies (Invitrogen, Carlsbad, CA) for 2 hours. Samples are washed with phosphate buffer and imaged. ROI were imaged using Nikon upright confocal microscope and were analyzed blindly for optical density using Image-J software. Negative control were treated with same protocol except for primary antibody.

### Pericytes immuno-localization studies

Eyes of diabetic animals were injected with an adeno-associated virus (AAV) with NG2 promoter that express green fluorescence protein (GFP) and Ephrin-B2 shRNA (ID: VB160513-1081jgm-pAAV[Exp]-NG2enhancerMinPro>EGFP:5'miR30:rEfnb2[shRNA#8]:3'miR30, Vector builder, Cyagen Biosciences Inc., Santa Clara, CA). 2 μl of purified viral particles (1× 10^11^ GC/ml) were injected using Hamilton syringe, 30-gauge needle, into the rat eye. Eyes were isolated 2 weeks after injection and fixed in 2% paraformaldehyde. Retinas were dissected and blocked using 0.1% horse serum dissolved in 1% BSA in 0.3% Triton X-100 in phosphate buffer. Retinas were reacted to Iso-lectin B4 (Invitrogen, Carlsbad, CA) and PDGFR-β, pericyte marker, (Abcam, Cambridge, MA). Immunohistochemical localization of GFP expression and pericyte marker are imaged using Nikon upright confocal microscope. PDGFR-β antibodies were specific for rat and used in 1:150 dilutions in 1% BSA in 0.3% Triton X-100 in phosphate buffer followed by incubation with 1:1000 dilutions of secondary conjugated goat anti-mouse (Invitrogen, Carlsbad, CA) for 2 hours.

### Novel object recognition

Novel object recognition (NOR) test was recorded 8 weeks after induction of diabetes. Before testing, rats were habituated in testing boxes in the absence of objects for two consecutive days. On the test day, a single rat was placed in the box for 30 min of free exploration training session in the presence of two equidistant and identical objects. To test short-term memory, after a 30 min break the rat was returned to the box with two objects, one of them was totally novel for 15 min of free exploration session. All sessions were video recorded and analyzed blindly. The time spent by each rat with its nose on each object was used to indicate the object preference. The D2 ratio index was calculated by dividing difference between time spent at the novel object and familiar object by the total time spent with the both object (in seconds).

### Cell culture

Human brain microvascular endothelial (HBMVE) and pericyt (HBMVP) cells were purchased from Angio-Proteomie (Boston, MA). Cells were grown in complete medium (Angio-Proteomie, Boston, MA). All experiments were performed in triplicates using cells between passages 4 and 6. Cells were switched to serum-free medium before cell migration assay or treatment application. Diabetic conditions for pericytes were performed via treatments with high glucose (25mM) and sodium palmitate (200 uM). Equimolar of L-glucose has been used as osmotic control. For pericytes/endothelial co-culture studies, trypsinized pericytes were mixed with endothelial cells in ratio 1 to 4 respectively.

### Cell migration

Cell migration was performed as described previously [20]. Briefly, BMVPCs were grown to form a monolayer and switched to serum free medium. The monolayer is then scratched with 1 ml pipette tip. Cell tracker (Invitrogen, Carlsbad, CA) is added to visualize cells. The scratches are imaged at zero time and after 18 hours. Percent migration was calculated and blotted as percent to control. Equimolar of L-glucose has been used as osmotic control.

### Tube formation

Tube formation assay was performed using growth factor-reduced Matrigel (BD Biosciences, Franklin Lakes, NJ) as described previously [21]. Briefly, BMVPCs were counted and plated over solidified Matrigel in a 4 well-chamber slide. After 24 hr., the formed tubes were imaged and analyzed. Results were represented as percentage to control. Equimolar of L-glucose has been used as osmotic control.

### Ephrin-B2 silencing

For cell migration studies and tube formation studies, BMVPC were transfected with either scrambled, Ephrin-B2 siRNA (Santa Cruz, Santa Cruz, CA) using Amaxa nucleofector kit according to the manufacturer’s protocol (Lonza, Koeln, Germany). For each zap, 0.5 million cells were re-suspended in a nucleofection solution with the siRNA and pmax-GFP then electroporated.

Transfected cells were left to recover in complete medium for 24 hours. Experiments were performed in triplicates within 72 hours after transfection. Transfection was confirmed by western blot and GFP expression. Transfected cells were left to recover in complete medium for 24 hours. Experiments were performed in triplicates within 72 hours after transfection.

### Western blot analysis

Brain/cells were homogenized in RIPA buffer (Millipore, Billerica, MA). Equal protein loads were mixed with Laemmli sample buffer and separated on a 4–15% gradient SDS-polyacrylamide gel by electrophoresis. Gels were then transferred to a nitrocellulose membrane and reacted with primary antibody. All primary antibodies were rat specific: polyclonal anti-Ephrin-B2 was purchased from (R&D systems; Minneapolis, MN), polyclonal phsopho-Ephrin-B2 and phospho-FAK were purchased from (Invitrogen; Rockford, IL), monoclonal anti-FAK was purchased from (Millipore), and monoclonal anti-actin was from (Sigma; St. Louis, MO). The membrane is then reacted with the proper horseradish peroxidase-conjugated secondary antibody. The membrane was reacted to Western chemiluminescent HRP Substrate (Millipore) and scanned over X-ray films. The films were subsequently developed, scanned, and band intensity was quantified by use densitometry Image-J software.

### Statistical analysis

**Fig 1:**
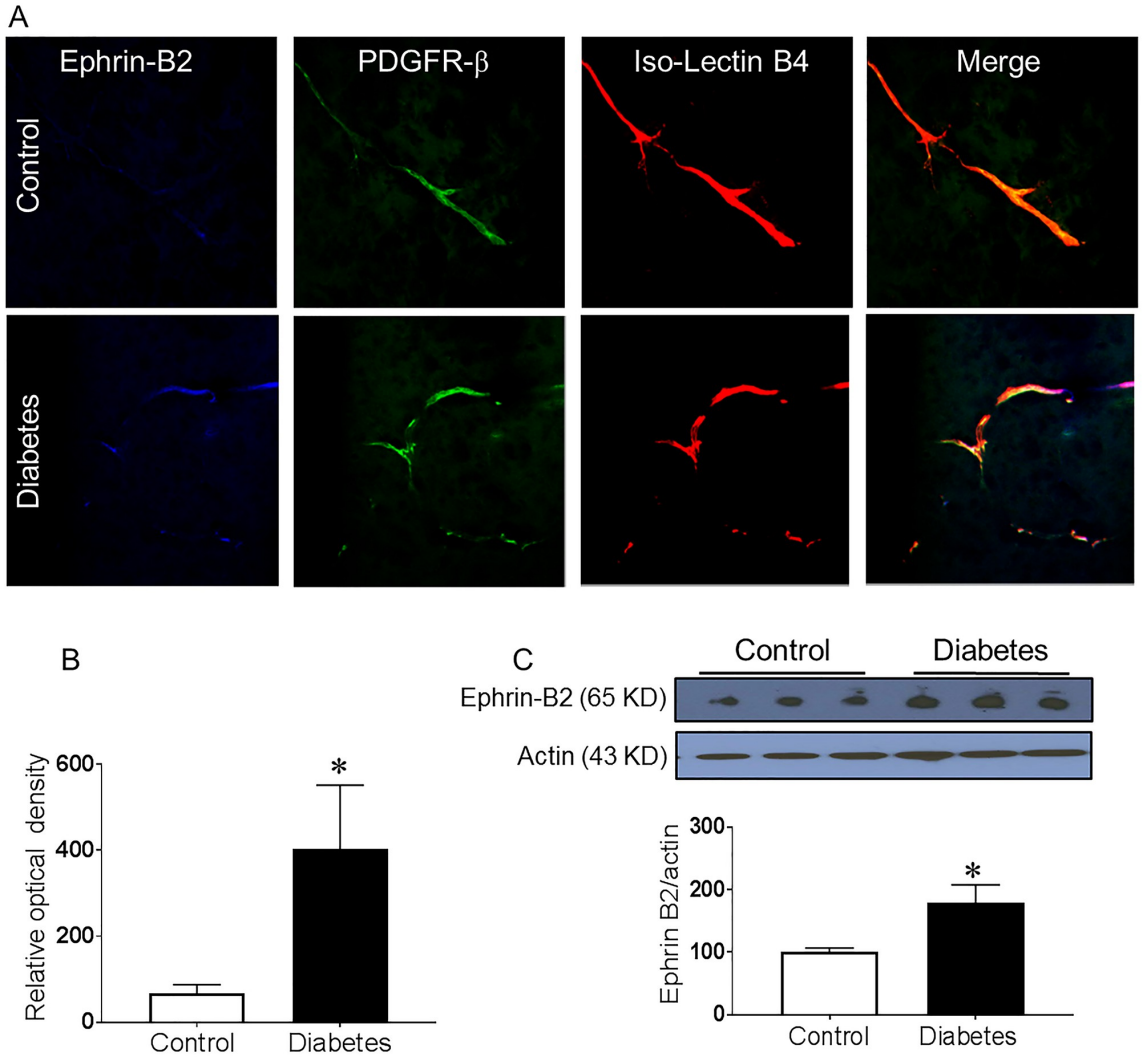
Increased Ephrin-B2 expression in pericytes in diabetes. Four weeks male Wistar rats were injected with low dose streptozotocin followed by 8-weeks of high-fat diet (HFD, 45% fat). Brains were isolated and fixed. Brain sections were reacted with anti-Ephrin-B2 antibody and Iso-Lectin-B4. (A) Co-localized Ephrin-B2 (Blue), PDGFR-β (green) on Iso-Lectin-B4 (red) was compared between diabetic and control Wistar rats. Diabetic rats showed increased Ephrin-B2 expression in perivascular area that was co-localized with the pericytes marker. (B) Quantification of the relative optical density. (C) Western blot analysis showed a significant increase in Ephrin-B2 expression in the brain cortex of diabetic rats compared to control. (N = 4–5, *P<0.05 vs control).

**Fig 2:**
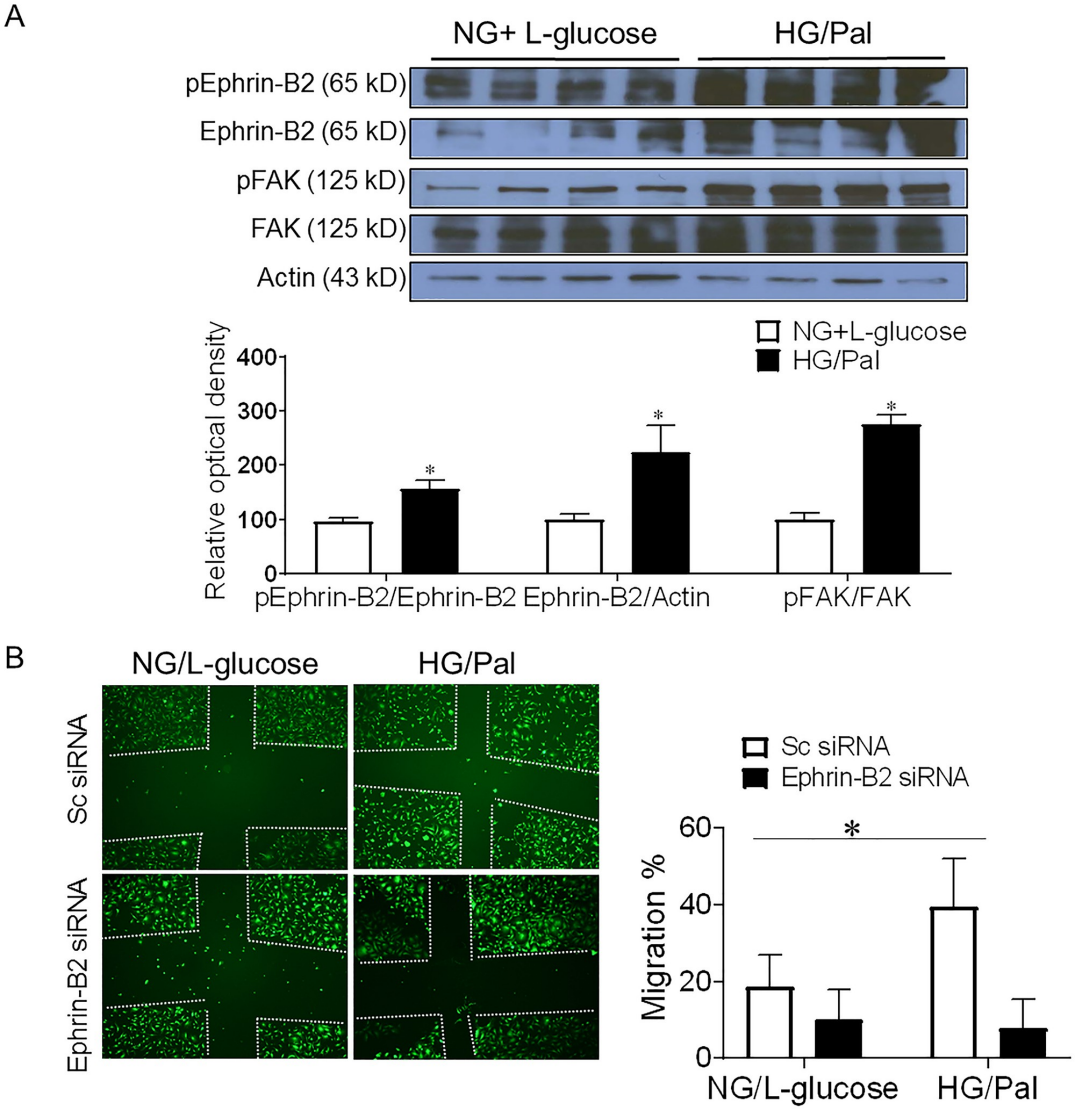
Diabetic conditions increases Ephrin-B2 signaling in pericytes. Human brain microvascular pericyte cells were treated with high glucose (25 mM) and sodium palmitate (200 uM) to mimic diabetic conditions. Equimolar of L-glucose was used as an osmotic control. A) Western blot analysis showed a significant increase in Ephrin-B2 expression and activation in pericytes under diabetic conditions. Moreover, results showed that diabetes caused an increase in downstream angiogenic signal as illustrated with enhanced focal adhesion kinase activation in pericytes. (*P<0.05 vs NG, n = 4). B) Pericytes were treated with high glucose (25mM glucose) and sodium palmitate (200 uM) showed 2 fold increase in cell migration assay. Silencing Ephrin-B2 was achieved using Ephrin-B2 siRNA. Silencing Ephrin-B2 in pericytes abolished diabetes-induced increase migration. (Two way ANOVA, Gene vs diabetic condition, significance: yes, Interaction: yes, n = 3 duplicate).

**Fig 3:**
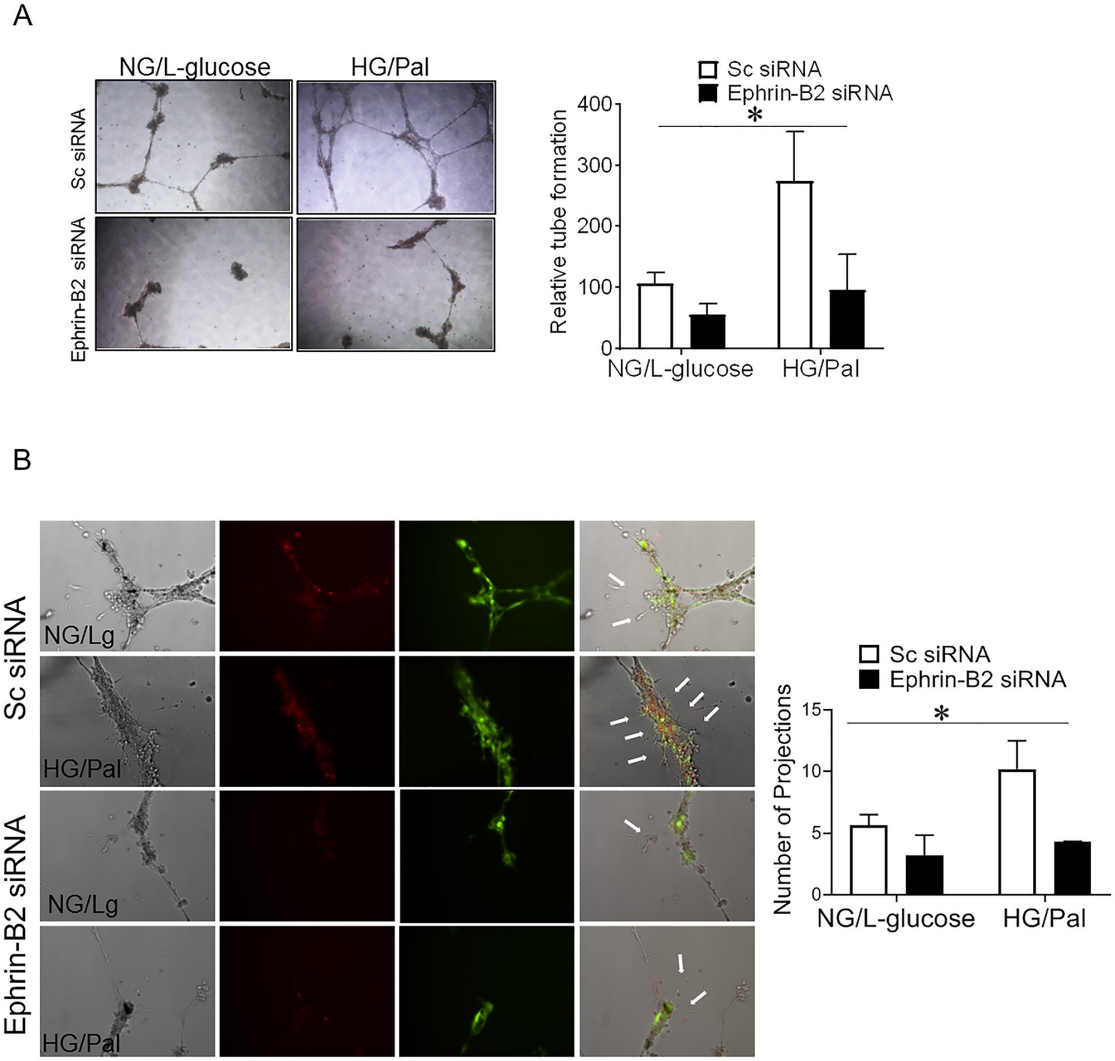
Silencing Ephrin-B2 expression in pericytes decreased diabetes-mediated angiogenic signaling. Ephrin-B2 was silenced in human brain microvascular pericytes using siRNA technology. Human brain microvascular pericytes and endothelial cells were grown in ratio one to four respectively in normal glucose or high glucose (25 mM) and palmitate (200 uM) to mimic diabetic conditions. L-glucose was used as an osmotic control. Representative images and quantifications from matrigel tube formation and filopodia extensions in pericytes/endothelial co-cultures are shown in panels A and B. Our results shows a significant increase in tube formation and number of filopodia extensions in diabetic conditions compared to control. Silencing Ephrin-B2 expression in pericytes abolished diabetes induced angiogenic signaling in pericytes/endothelial cell co-culture. (Two way ANOVA, Gene vs diabetic condition, significance: yes, Interaction: yes, n = 3 duplicate).

**Fig 4:**
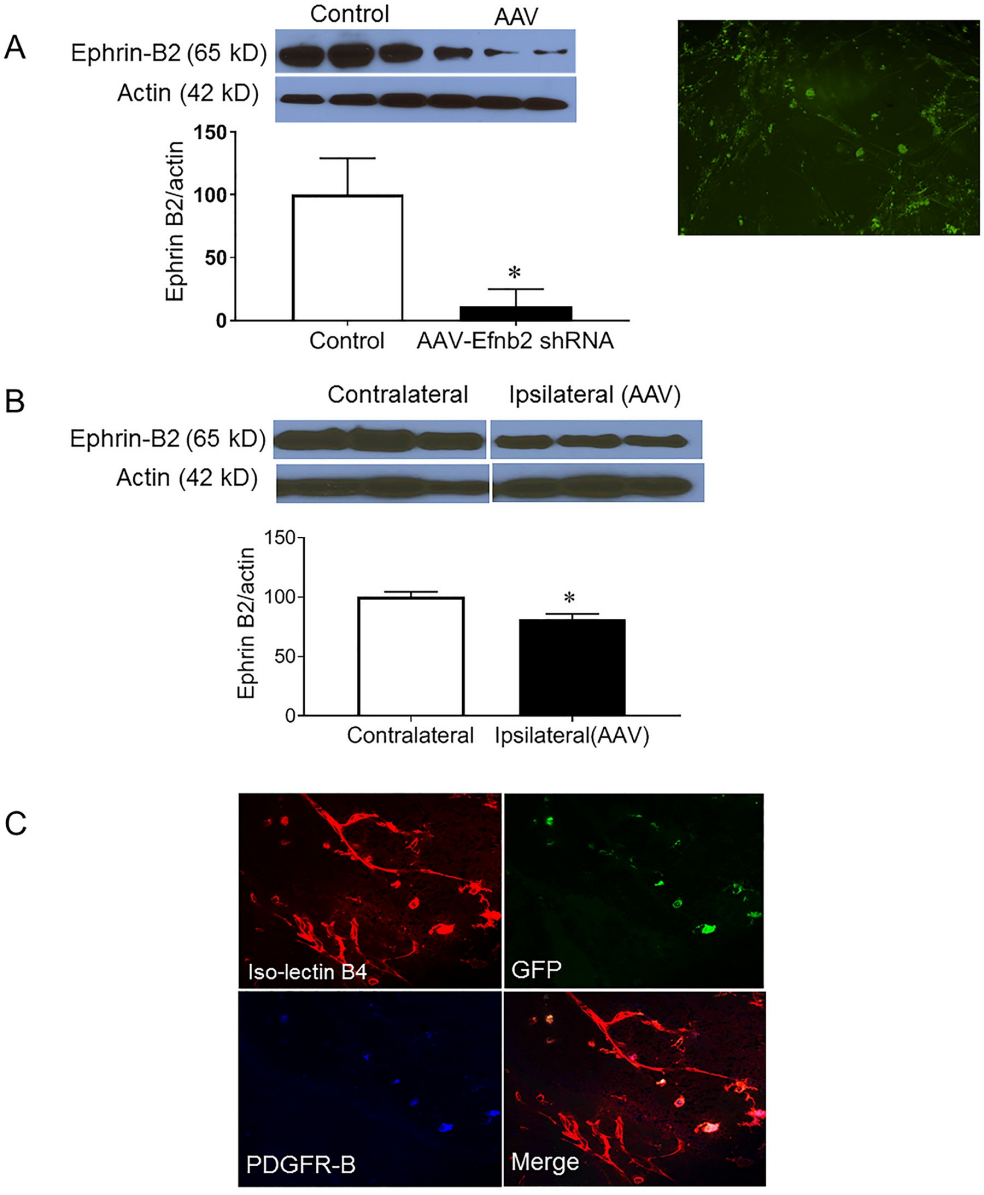
Selective reduction of Ephrin-B2 expression in pericytes in vivo. A) Ephrin-B2 expression was significantly reduced in human brain microvascular pericytes using adeno-associated virus (AAV) the express Ephrin-B2 shRNA under NG-2 promotor, a pericyte promotor. Immunoblotting studies confirmed significant reduction of Ephrin-B2 in pericytes. Pericytes expressed green fluoresce protein as another indication of successful transfection. (*P<0.05 vs Control, n = 3). (B) Immunoblotting studies showed a decrease in Ephrin-B2 expression in ipsilateral hemisphere after stereotactic injection of the AAV into striatum compared to the contralateral side. (*P<0.05 vs contralateral, n = 3). (C) Representatives of immunohistochemical studies showing the localization of the AAV green fluoresce protein expression with only the pericyte marker, PDGFR-β, but not the endothelial cell marker, iso-lectin B4 following intraocular injection of the AAV virus.

**Fig 5:**
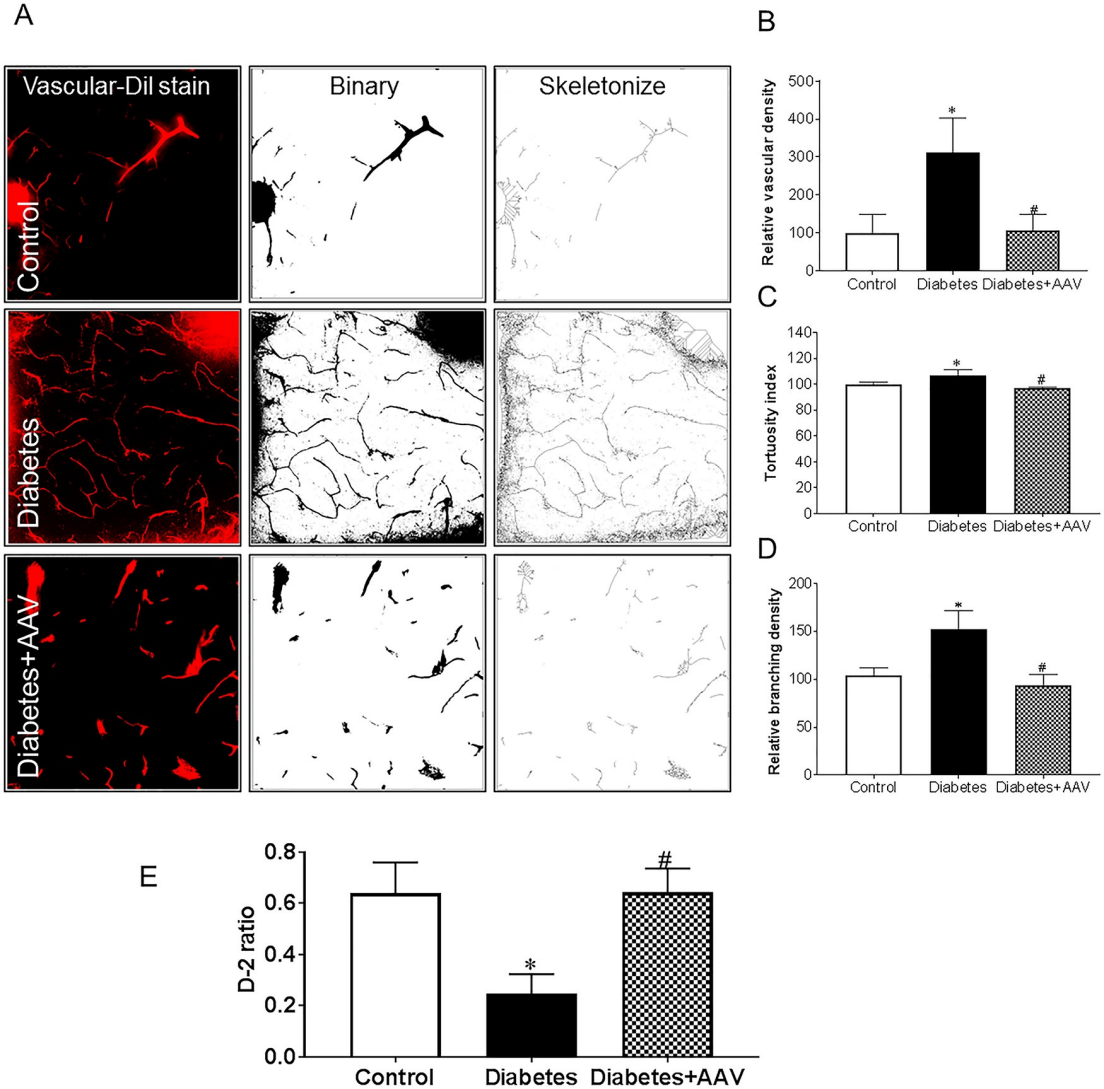
Silencing Ephrin-B2 in pericytes in diabetic rats restored cerebral vascularization and improved cognitive functions. Ephrin-B2 was decreased in cerebral pericytes using AAV viral injection. Animals were perfused with vascular Dil stain. (A) Representatives of brain sections imaged using confocal microscopy. 3D images reconstructs were quantified for neovascularization indices (B) vascular density (C) tortuosity and (D) branch density. Diabetes-induced pathological neovascularization, and selective inhibition of Ephrin-B2 in pericytes restored the neovascularization indices in diabetic rats. E) Novel object recognition test showing cognitive deterioration of diabetic animals compared to control. Selective inhibition of Ephrin-B2 in pericytes improved cognitive functions in diabetes. (N = 4–6, *P<0.05 vs control, ^#^P<0.05 vs diabetes).

Statistical significance for all analyses was assessed at an alpha level of 0.05 using GraphPad Prism (GraphPad Inc., version 7.04). A Tukey-Kramer adjustment for multiple comparisons is used for all post-hoc mean comparisons. One-way ANOVA was used to compare the means of groups in Figs 1, 2A, 4 and 5. Two-way ANOVA was used to compare groups in the tube formation and number of the projections in Figs 2B and 3. Results were presented by mean ± standard error.

## Results

### Increased Ephrin-B2 expression in diabetes

Ephrin-B2 expression was examined in cerebrovasculature of control and type II diabetic rats using immunohistochemistry and immunoblotting. Our results showed a significant increase in Ephrin-B2 expression in the perivascular areas in the brain cortex that is co-localized with pericytes (Fig 1A and 1B).

### Increased Ephrin-B2 expression enhanced pericyte angiogenic signaling

Culturing human brain microvascular pericytes in diabetes-mimicking conditions (high glucose and palmitate) increased Ephrin-B2 expression and activity. Moreover, our results showed an increase in downstream angiogenic signaling as shown by increase in activation of focal adhesion kinase (Fig 2A). Pericytes migration was significantly increased (~2-fold) following the treatment with diabetic conditions compared to control, this effect was attenuated by silencing Ephrin-B2 (Fig 2B).

### Silencing Ephrin-B2 expression in pericytes decreased diabetes-induced neovascularization

Pericytes/endothelial co-culture exposed to diabetic conditions (high glucose and palmitate) treatment showed an increase in tube formation in Matrigel assay compared to control. Silencing Ephrin-B2 in pericytes significantly reduced the ability of pericytes/endothelial co-culture to form tubes (Fig 3A). Since filopodial extensions, direction of endothelial tip cells are important in the migration of the endothelial cells, we investigated the impact of Ephrin-B2 silencing on the number of filopodia. We found that the number of filopodia extensions was significantly upregulated in the pericyte/endothelial co-culture following the treatment with high glucose/palmitate. Silencing Ephrin-B2 in pericytes decreased the number of filopodia extensions of endothelial cells under diabetic conditions (Fig 3B). Therefore, increased angiogenic properties under diabetic conditions was significantly reduced after silencing Ephrin-B2 in pericytes.

### Selective reduction of Ephrin-B2 expression in pericytes in vivo

AAV expressing Ephrin-B2 shRNA with NG2 promoter, a pericyte promotor, was injected into striatum of diabetic animal using a stereotactic injection. Immunoblotting and immunohistochemical studies confirmed successful inhibition of Ephrin-B2 expression in human brain microvascular pericytes cells (Fig 4A). Stereotactic injection of the AAV into striatum showed a small but significant decrease of Ephrin-B2 expression when compared to contralateral side (Fig 4B). The retina is known as an extension of the CNS and it shares plenty of cerebrovascular properties. The accessibility of the retina, unlike the brain, has provided an opportunity to use the retinal vasculature to validate the use of AAV in targeting the pericytes. Intraocular injection of the AAV virus showed a localization of the AAV GFP expression with only the pericyte marker, PDGFR-β, but not the endothelial cell marker, isolectin B4 (Fig 4C).

### Pericyte-conditional Ephrin-B2 inhibition decreased diabetes induced pathological neovascularization and improved vascular integrity

We have previously shown that diabetes caused a significant increase in cerebral pathological neovascularization [4, 20, 22]. Our current study showed that the selective inhibition of Ephrin-B2 in pericytes restored the neovascularization indices (vascular density, tortuosity and branch density) in diabetic rats, (Fig 5A–5D). Moreover, our results showed that inhibition of pathological neovascularization was also accompanied with an enhancement of the neurocognitive functions of diabetic rats reflected in the improvement of D2 ratio by the novel object recognition test (Fig 5E).

## Discussion

**Fig 6:**
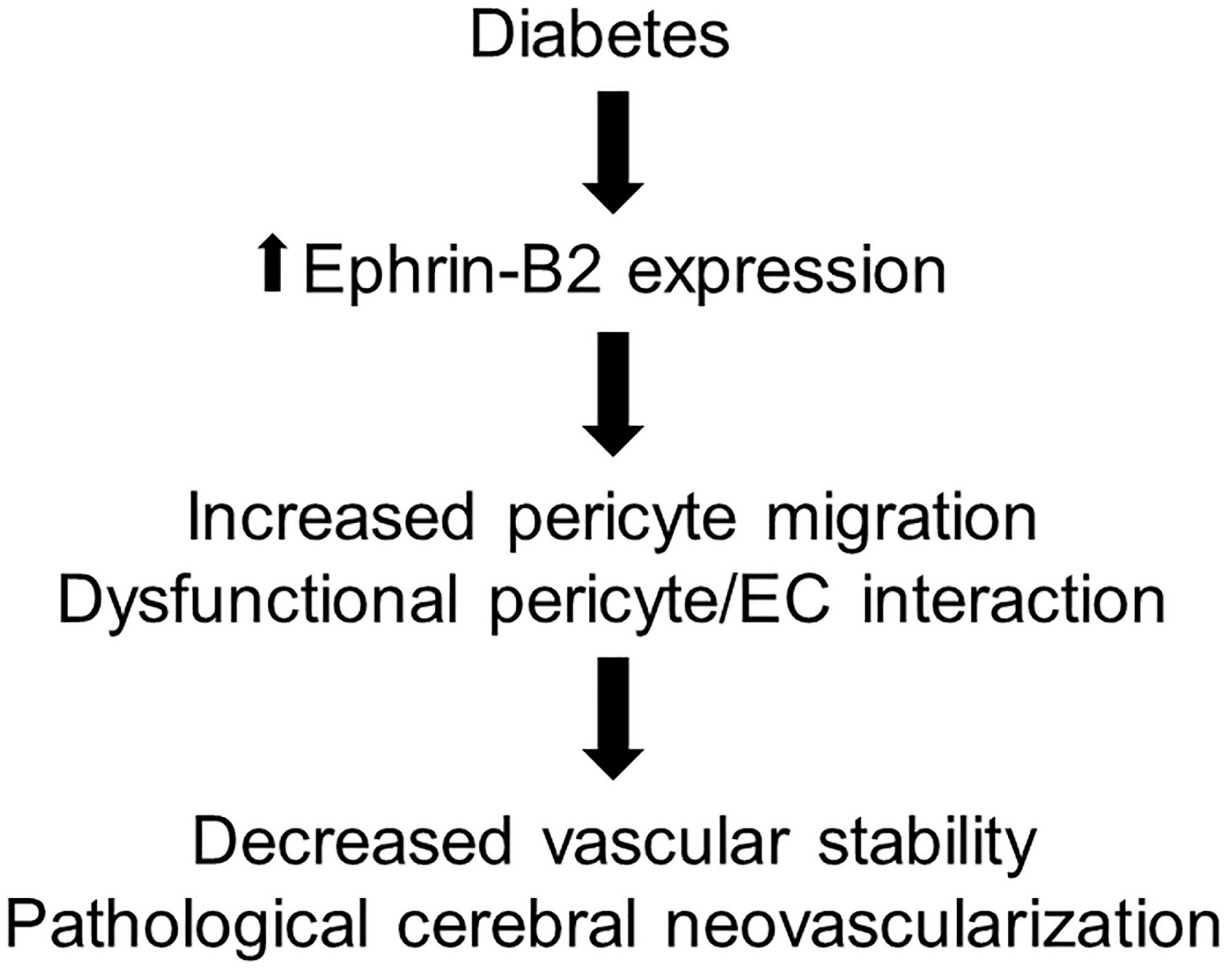
Increased Ephrin-B2 signaling contributes to diabetes-induced cerebral pathological neovascularization. Increased Ephrin-B2 signaling in pericytes in diabetes enhances pericytes migration and impairs pericytes/endothelial cells interactions leading to a decrease in vascular stability and pathological cerebral neovascularization.

In the current study, we present new evidence that pericytes contribute to diabetes-induced cerebral pathological neovascularization. We show that diabetes enhances Ephrin-B2 in pericytes which disturbs pericyte/endothelial interaction and increases cerebral neovascularization. Suppression of EphrinB-2 expression in pericytes decreases angiogenic properties, restores cerebral vascularization and improve cognitive functions in diabetes. Altogether, our findings emphasize the crucial role of pericytes in the pathological neovascularization in diabetes and establish Ephrin-B2 signaling as a potential target to improve cerebrovascular integrity in diabetes (Fig 6).

We have previously shown increased cerebral neovascularization in different animal models of type II diabetes such as Goto-Kakizaki rats and db/db mice [23]. These studies revealed that upregulated expression of vascular endothelial growth factor increased endothelial cells misguidance into newly formed micro vessels that are unorganized, poorly perfused and leaky [5, 20, 23]. In the present study, we detected the same phenomenon in another well-established rat model of type II diabetes, high fat diet/small dose streptozotocin (HFD/STZ). We demonstrated an increase in vascular density, tortuosity and branching density in diabetic rats. Yet, the current study focuses on the role of another member of the neurovascular unit, the pericytes.

Pericytes share the basal membrane and the first to interact with the endothelial cells in the microvasculature [10]. Pericyte to endothelial ratio is highest in the cerebral microvasculature when compared with other vascular beds. Pericytes are pivotal for stabilizing the integrity and preventing the leakage of the cerebral microvasculature via regulating the tight junctions and endothelial permeability [24]. In addition, pericytes control action of the proteases on the extra cellular matrix, via expression of tissue inhibitor of metalloproteinase-3 [13]. Taken together, all this, account for how pericytes tunes up endothelial cell guidance and angiogenesis process [11, 14, 25].

Pericytes are vulnerable cells that die early in diabetes as well as other diseases [10, 25]. Diabetes decreases the number of pericytes in the dermal and muscle capillaries in diabetic patients [26]. Pericyte loss is considered a hallmark of early diabetic retinopathy that aggravates the disease into proliferative stage [27]. Additionally, reduced pericyte number in brain arteriovenous malformations contributes to vascular fragility and increased vascular leakage [25]. Yet, the role of pericytes in diabetic-induced cerebral microangiopathy remains unclear. Having knowledge of the mechanism of diabetes-induced cerebral microangiopathy will advance our therapeutic strategies to control cerebral complication in diabetes.

Ephrin-B2 and its receptor EphB4, members of Ephrin/Eph tyrosine kinase receptors family, are involved in cell-cell communication, endothelial guidance during development and postnatal angiogenesis [16–18]. Ephrin-B2 is expressed in both endothelial cells and pericyte cells [18, 28]. Increased expression of Ephrin-B2 has been reported in conditions associated with increased pathological angiogenesis such as proliferative diabetic retinopathy and tumor growth [15, 29, 30]. Yet, how diabetes alters Ephrin-B2 expression in pericytes in cerebrovasculature is a gap in our knowledge.

In the current study, we tested the hypothesis that diabetes causes an increase in Ephrin-B2 expression in pericytes that contributed to diabetes-mediated pathological cerebral neovascularization. To test our hypothesis, we used a rat model of type II diabetes, HFD/STZ. Our results showed that diabetes-induced pathological cerebral neovascularization was accompanied with an increased Ephrin-B2 expression in the diabetic rat cerebrovasculature compared to control.

To further test our hypothesis, we exposed human microvascular pericytes to high glucose and palmitate to mimic diabetic condition. Our results showed an increased Ephrin-B2 expression in brain pericytes which was accompanied with an enhanced downstream angiogenic signal. We reported an upregulated activity of the downstream migratory kinases as focal adhesion kinase, FAK. These results came in agreements with studies that showed an increased Ephrin-B2 expression in proliferative angiogenesis as in diabetic retinopathy, after cerebral ischemia and tumors [15, 29–31]. On the other hand, we showed that silencing Ephrin-B2 in pericytes decreased diabetes-induced angiogenic properties as seen in decreased pericyte migration and pericyte/endothelial cell co-culture reduced tube formation. These results were also confirmed with *in vivo* studies where we achieved selective decrease in Ephrin-B2 expression in pericytes via stereotactic injection of AAV particles that express Ephrin-B2 shRNA under NG2 promoter, pericyte marker. Our data showed that selective inhibition of Ephrin-B2 in pericytes significantly decreased the diabetes-induced cerebral pathological neovascularization indices. In parallel, when vascular integrity was improved by selective inhibition of Ephrin-B2 expression in cerebrovascular pericytes, cognitive function of the diabetic animals also improved.

In summary, the current study reveals the crucial role of pericytes in the diabetes-induced pathological cerebral neovascularization. In addition, our findings greatly accentuate Ephrin-B2 signaling as a promising therapeutic target to improve cerebrovascular integrity in diabetes. Further studies addressing the role and mechanisms by which the Ephrin-B2 receptor, EphB4, regulate pericytes/endothelial interaction in diabetes are needed to better understand diabetes-induced pathological cerebral neovascularization.
